# Serum screening in first trimester to predict pre-eclampsia, small for gestational age and preterm delivery: systematic review and meta-analysis

**DOI:** 10.1186/s12884-015-0608-y

**Published:** 2015-08-25

**Authors:** Yan Zhong, Fufan Zhu, Yiling Ding

**Affiliations:** The Second Xiangya Hospital, Central South University, No.139, Middle Renmin Road, Changsha, Hunan 410011 P.R. China

## Abstract

**Background:**

Early assessment before the establishment of placental dysfunction has the potential to improve treatment and prognosis for clinical practice.The objective of the study is to investigate the accuracy of serum biochemical markers(Pregnancy- Associated Plasma Protein-A (PAPP-A), human Chorionic Gonadotropin (hCG), Placental Growth Factor (PlGF), Placental Protein 13 (PP13) used in first trimester serum screening in predicting preelampsia, small for gestational age (SGA) and preterm delivery.

**Methods:**

The data sources included Medline, Embase, Cochrane library, Medion, hand searching of relevant journals, reference list checking of included articles and contact with experts. Two reviewers independently selected the articles. Two authors independently extracted data on study characteristics, quality and results.

**Results:**

The results showed low predictive accuracy overall. For preeclampsia, the best predictor was PlGF; LR + 4.01 (3.74, 4.28), LR-(0.67, 0.64, 0.69). The predictive value of serum markers for early preeclampsia was better than that of late preeclampsia. For SGA the best predictor was PP13; LR+ 3.70 (3.39, 4.03), LR- 0.70 (0.67, 0.73). For preterm delivery, the best predictor was PP13; LR+ 4.16 (2.72, 5.61), LR- 0.56 (0.45, 0.67).

**Conclusion:**

First trimester screening analytes have low predictive accuracy for pre-eclampsia, small for gestational age and preterm delivery. However, the predict value of first trimester analytes is not worse than that of the second trimester markers.

## Background

Preeclampsia, fetal growth restriction (FGR) and preterm delivery are major contributors to perinatal mortality and morbidity. They not only alter the immediate outcomes of pregnancy at the time of delivery but also the long-term cardiovascular health of the affected women and children. For example, a history of preeclampsia increases a female’s risk of myocardial infarction, stroke or diabetes mellitus by two to eight fold over the next two decades [1]. Moreover, newborns diagnosed with FGR at birth have a two to eight fold increased risk for hypertension, cardiovascular disease, diabetes mellitus or renal disease as adults [2, 3].

Recent evidence suggests that the underlying pathology of preeclampsia, FGR and preterm delivery takes place in the first trimester. Earlier assessment before the establishment of placental dysfunction may have the potential to improve treatment and prognosis for clinical practice. Numerous stutdies have shown that abnormal concentration of first trimester serum markers is related to the onset of preeclampsia, small for gestational age and preterm delivery. With the increased use of first-trimester screening for Down syndrome, there is the opportunity to ‘piggy back’ screening tests for preeclampsia, FGR and preterm delivery onto existing tests.

The purpose of our review was to investigate the accuracy of serum biochemical markers (Pregnancy- Associated Plasma Protein-A (PAPP-A), human Chorionic Gonadotropin (hCG), Placental Growth Factor (PlGF), Placental Protein 13 (PP13) used in first trimester serum screening in predicting preelampsia, small for gestational age (SGA) and preterm delivery. We systematically reviewed the available literature and meta-analysed the data.

## Methods

### Identification of studies

We searched MEDLINE, EMBASE and Cochrane Library from inception to April 2014 for relevant citations. The reference lists of all known primary and review articles were examined to identify cited articles not captured by electronic searches. The search strategy consisted of MeSH (medical subject heading) terms, Emtree terms, and keywords related to the disease (preeclampsia, small for gestational age, preterm birth, preterm delivery, etc.) combined with serum markers(PAPP-A, hCG, PP13, PlGF, etc.). Details of the search strategy are available from the authors. Language restrictions were not applied. A comprehensive database of relevant articles was constructed.

### Study selection

The first stage of study selection was the scrutinizing of the database by two reviewers to identify articles from title and/or abstract. In a second stage, a search based on keywords for each of the analytes under review was performed within the Reference Manager database. The results of this search were scrutinized by a second reviewer. In the final stage of study selection the full papers of identified articles were obtained with final inclusion or exclusion decisions made after independent and duplicate examination of the papers. We included studies that reported on singleton pregnancies at low risk in any healthcare setting before the 14^th^ week of gestation. Test accuracy studies allowing generation of 2 × 2 tables were included.

### Data extraction and study quality assessment

Acceptable reference standards for preeclampsia were: persistent systolic blood pressure ≥ 140 mmHg or diastolic blood pressure ≥ 90 mmHg with proteinuria ≥ 0.3 g/24 h or ≥ 1+ dipstick (= 30 mg/dl in a single urine sample), new after 20 weeks of gestation. Early preeclampsia was defined as preeclampsia resulting in a delivery before 34 weeks of gestation. Late preeclampsia was defined as preeclampsia resulting in a delivery after 34 weeks of gestation. Acceptable reference standards for SGA included birth weight < 10^th^ centile adjusted for gestational age and based on local population values. We also included severe SGA defined as birth weight < 5^th^ centile. Preterm delivery was defined as delivery < 37 weeks. We also included preterm delivery < 34 weeks.

All included manuscripts were assessed by at least one reviewer for study and reporting quality using validated tools. Items considered important for a good quality paper were prospective design with consecutive recruitment, prospective design, adequate description of selection criteria, patient spectrum,test and use of appropriate reference standard.

### Data synthesis and analysis

From the 2 ×2 tables the following were calculated with their 95 % confidence intervals for individual studies; sensitivity (true positive rate), specificity (true negative rate) and the likelihood ratios (LR). Results were pooled among groups of studies with similar characteristics, the same threshold and same adverse outcomes. Where 2 × 2 tables contained zero cells, 0.5 was added to each cell to enable calculations. All statistical analyses were performed using Stata 11.0 statistical package.

## Results

### Literature identification and study quality

**Fig. 1:**
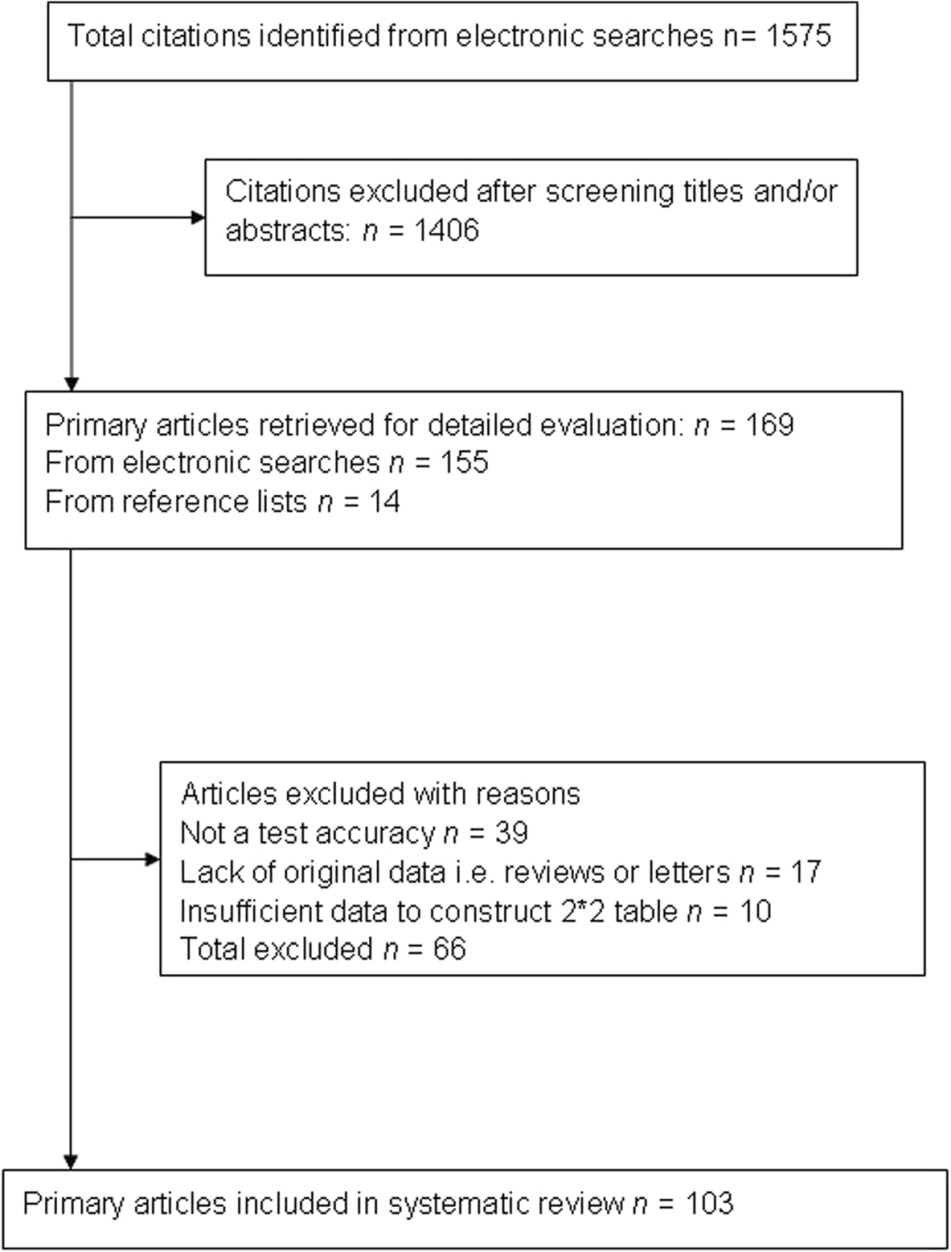
Seletion process: process from initial search to final inclusion for biochemical screening to predict pre-eclampsia/small for gestational age/preterm delievery

Figure 1 summarises the process of literature identification and selection. There were 1575 primary articles that met the selection criteria. The initial electronic search strategy led to screen titles and abstracts of 1406 citations. Fig. 1 shows the screening and selection process that was followed for the identification and inclusion of studies. We retrieved 155 potentially eligible primary studies for detailed evaluation and inclusion in the systematic review, and an additional 14 potentially eligible publications from the reference lists of included studies. Detailed evaluation led to the exclusion of 66 publications that did not meet the selection criteria. Overall, 103 studies were considered relevant and were included in the systematic review. Total number of women in 103 studies is 432,621.

**Fig. 2:**
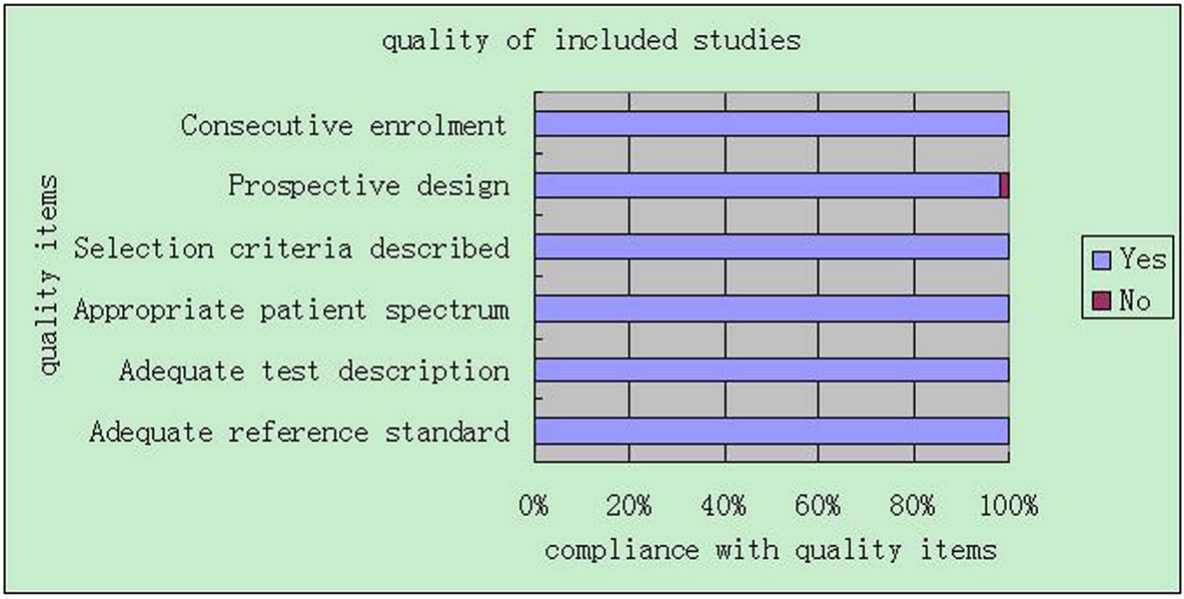
Study quality: bar chart showing quality of evidence on first trimester biochemical screening markers to predict pre-eclampsia, small for gestational age and preterm delivery

The quality assessment of included studies is summarized in Fig. 2. There was good reporting of prospective design with consecutive recruitment, prospective design, adequate description of selection criteria, patient spectrum, test and use of appropriate reference standard.

### Data analysis

For both analysis for preelampsia, SGA and preterm delivery, there was significant heterogeneity in all results. As a consequence of this the random effects model was used throughout the study.

### Pregnancy associated plasma protein A (PAPP-A)

**Fig. 3:**
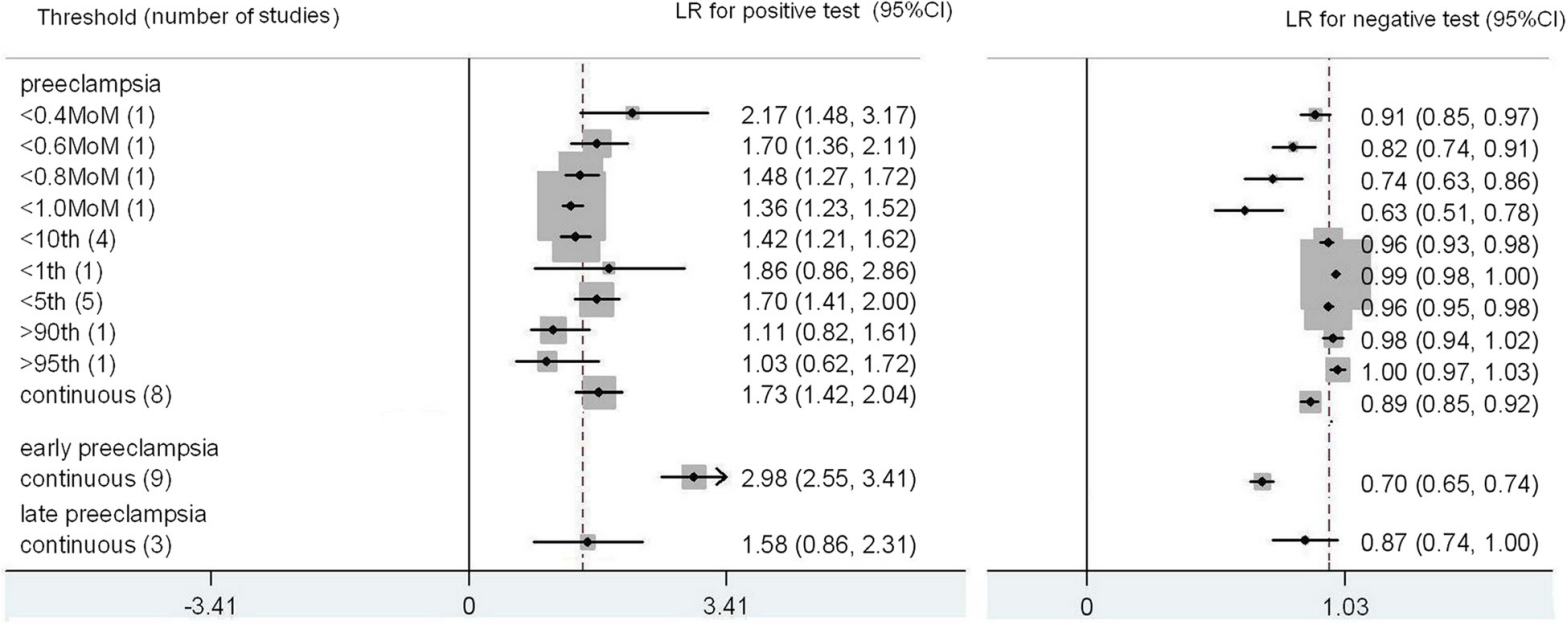
Forest plot for PAPP-A to predict preeclampsia: forest plot showing likelihood ratio of a positive and negative test result with 95 % confidence intervals (95 % CI) for studies of Pregnancy- Associated Plasma Protein-A (PAPP-A) to predict pre-eclampsia. * continuous: Likelihood ratio calculated from receiver operating curve analysis

The results for PAPPA to predict preeclampsia are summarized in Fig. 3. The total number of women in these studies is 385,643. There were 16 studies for preeclampsia, 10 for early preeclampsia and 3 for late preeclampsia included in the meta-analysis [4–22]. For preeclampsia, thresholds that were most commonly used were <5^th^ centile (5 studies) and < 10^th^ centile (4 studies). The most accurate predictor was PAPPA < 0.4 MoM (multiples of median); LR+ 2.17 (1.48,3.17), LR- 0.91 (0.85, 0.97), sensitivity 16 % (4 %, 35 %), specificity 93 % (76 %, 99 %), this was a single study. For early preeclampsia, all the predictive value was calculated from receiver operating curve analysis. The predictive value for early preeclampsia (LR+ 2.98 (2.55,3.41), LR-0.70 (0.65, 0.74), sensitivity (39 % (33 %, 47 %)), specificity (87 % (82 %, 90 %)) was generally better than that for preeclampsia and late preeclampsia (LR+ 1.58 (0.86, 2.31), LR-0.87 (0.74, 1.00), sensitivity 29 % (28 %, 30 %), specificity 82 % (81 %, 83 %)).

**Fig. 4:**
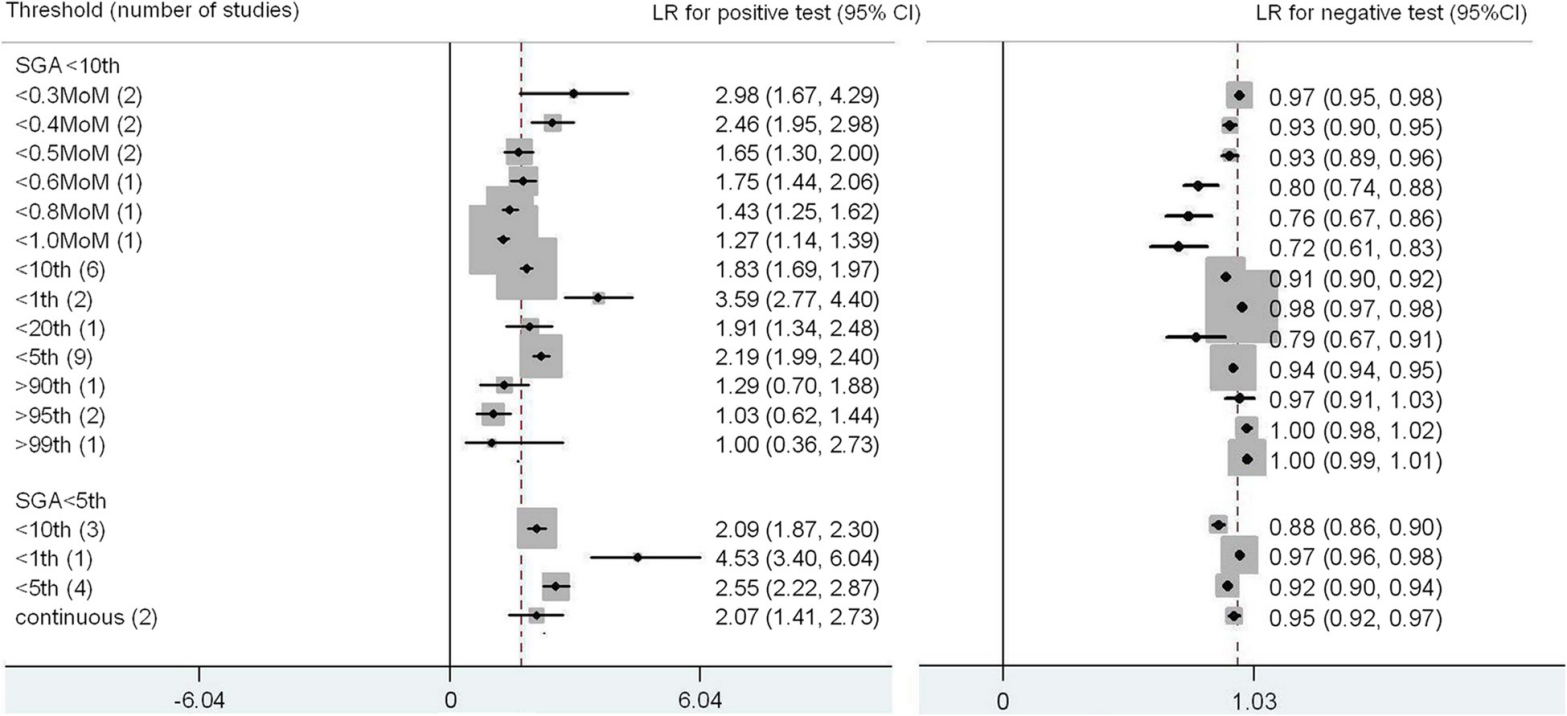
Forest plot for PAPP-A to predict SGA: forest Plot showing likelihood ratio of a positive and negative test result with 95 % confidence intervals (95 % CI) for studies of Pregnancy- Associated Plasma Protein-A (PAPP-A) to predict small for gestational age (SGA). * continuous: Likelihood ratio calculated from receiver operating curve analysis

For SGA there were 19 studies for SGA < 10^th^ centile and 6 studies for SGA < 5^th^ centile included in the meta-analysis [4, 6, 7, 10, 11, 23–36] (Fig. 4). The commonest threshold used to predict SGA < 10^th^ centile were PAPPA < 5^th^ centile (9 studies). The best predictor for SGA <10^th^ centile was PAPPA < 1^st^ centile; LR+ 3.59 (2.77, 4.40), LR- 0.98 (0.97, 0.98), sensitivity 3 % (3 %, 4 %), specificity 99 % (98 %, 99 %). For SGA < 5^th^ centile, PAPPA < 1^st^ centile was also the most accurate predictor, LR+ 4.53 (3.40, 6.04), LR-0.97 (0.96, 0.98), sensitivity 4 % (2 %, 5 %), specificity 99 % (98 %, 100 %).

**Fig. 5:**
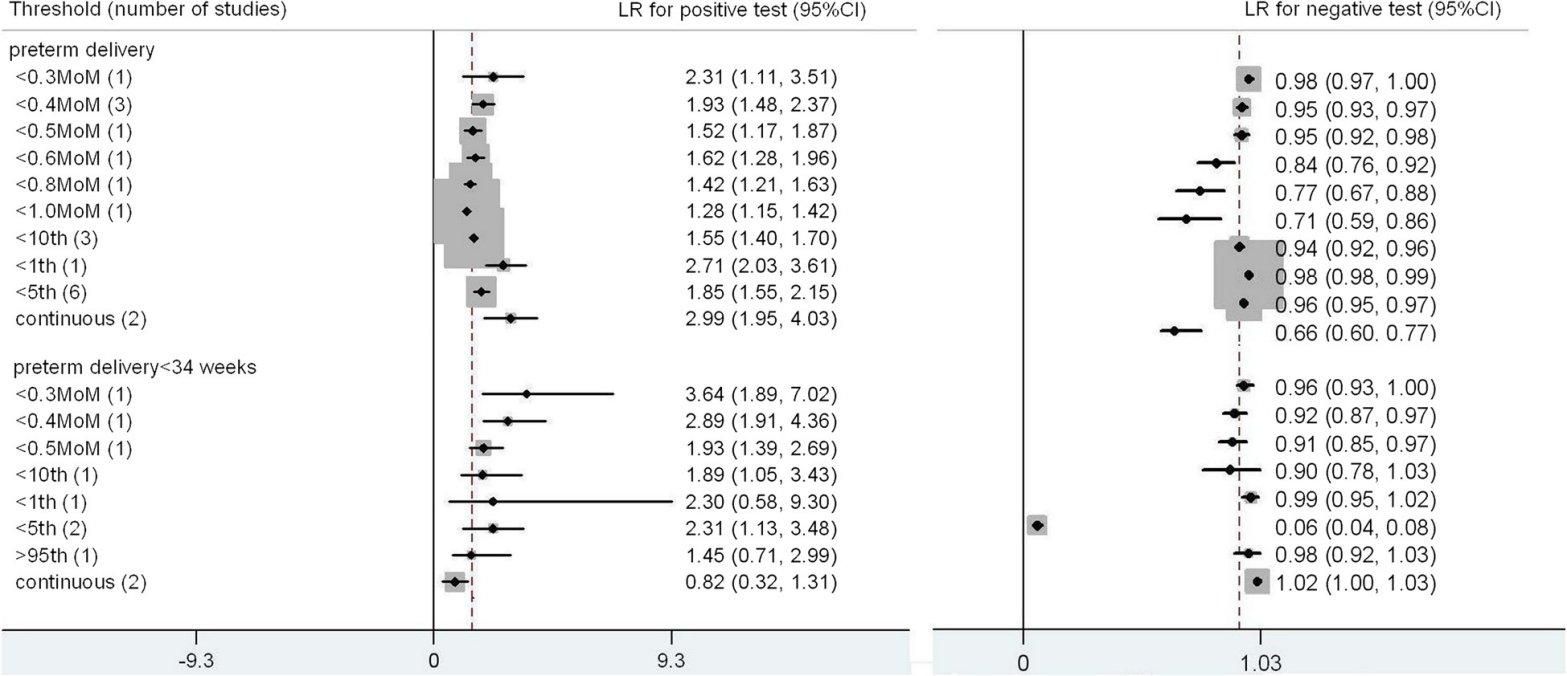
Forest plot for PAPP-A to predict preterm delivery: forest Plot showing likelihood ratio of a positive and negative test result with 95 % confidence intervals (95 % CI) for studies of Pregnancy- Associated Plasma Protein-A (PAPP-A) to predict preterm delivery. * continuous: Likelihood ratio calculated from receiver operating curve analysis

For preterm delivery there were 12 studies for preterm delivery and 6 studies for preterm delivery < 34 weeks included in the meta-analysis [4, 7, 10, 11, 23-25, 33, 35, 37–40] (Fig. 5). The commonest threshold used to predict preterm delivery were <5^th^ centile (6 studies). The best predictor for preterm delivery was calculated from receiver operating curve analysis; LR+ 2.99 (1.95, 4.03), LR- 0.66 (0.60, 0.77), sensitivity 44 % (28 %, 58 %), specificity 85 % (70 %, 93 %). For preterm delivery < 34 weeks, PAPPA < 0.3 MoM was the most accurate predictor, LR+ 3.64 (1.89, 7.02), LR-0.96 (0.93, 1.00), sensitivity 5 % (1 %, 14 %), specifcity 99 % (93 %, 100 %).

### Plancenta protein 13 (PP13)

**Fig. 6:**
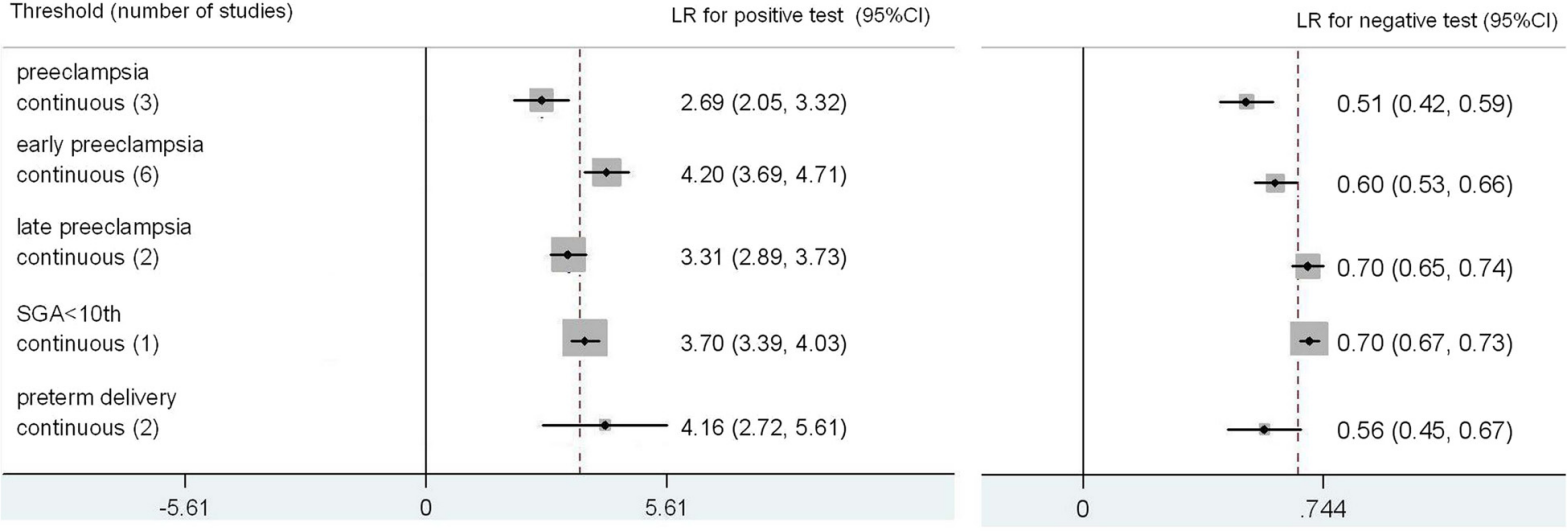
Forest plot for PP13 to predict preeclampsia, SGA and preterm delivery: forest Plot showing likelihood ratio of a positive and negative test result with 95 % confidence intervals (95 % CI) for studies of Placental Protein 13 (PP13) to predict preeclampsia, small for gestational age (SGA) and preterm delivery. * continuous: Likelihood ratio calculated from receiver operating curve analysis

The results for PP13 are summarized in Fig. 6, all the predictive value was calculated from receiver operating curve analysis. The total number of women in these studies is 60,786. For early preeclampsia there were 6 included studies [8, 12, 15, 41-44]. PP13 turns out to be more accurate predictor for early preclampsia; LR+ 4.20 (3.69, 4.71), LR-0.60 (0.53, 0.66), sensitivity 47 % (39 %, 54 %), specificity 89 % (85 %, 91 %). For preeclampsia there were 4 studies[8, 21, 45, 46]; LR+ 2.69 (2.05, 3.32), LR- 0.51 (0.42, 0.59), sensitivity 60 % (50 %, 73 %), specificity 78 % (64 %, 85 %). For SGA <10^th^ there was only one included study[29], LR+ 3.70 (3.39, 4.03), LR- 0.70 (0.67, 0.73), sensitivity 36 % (33 %, 41 %), specificity 90 % (88 %, 92 %). For preterm delivery, there were 2 included studies [39, 45]; LR+ 4.16 (2.72, 5.61), LR- 0.56 (0.45, 0.67), sensitivity 51 % (37 %, 66 %), specificity 88 % (76 %, 93 %).

### Placental growth factor (PlGF)

**Fig. 7:**
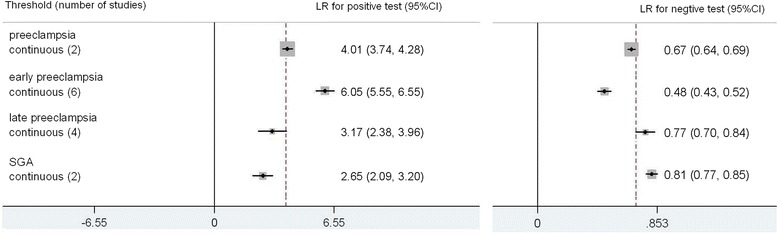
Forest plot for PlGF to predict preeclampsia, SGA and preterm delivery: forest Plot showing likelihood ratio of a positive and negative test result with 95 % confidence intervals (95 % CI) for studies of Placental Growth Factor (PlGF) to predict preeclampsia, small for gestational age (SGA) and preterm delivery. * continous: Likelihood ratio calculated from receiver operating curve analysis

The results for PlGF are summarized in Fig. 7. There were 16 included studies. The total number of women in these studies is 84,424. For preeclampsia there were 2 studies [13, 20]; LR + 4.01(3.74, 4.28), LR-0.67 (0.64, 0.69), sensitivity 40 % (37 %, 43 %), specificity 90 % (88 %, 91 %). PlGF was also shown to be more predictive for early preeclampsia[12-14, 17, 19, 20]; LR + 6.05 (5.55, 6.55), LR- 0.48 (0.43, 0.52), sensitivity 56 % (52 %, 61 %), specificity 91 % (89 %, 92 %). For SGA there were 2 included studies [29, 32]; LR+ 2.65 (2.09, 3.20), LR-0.81 (0.77, 0.85), sensitivity 27 % (20 %, 36 %), specificity 90 % (83 %, 94 %).

### Human chorionic gonadotrophin (hCG)

**Fig. 8:**
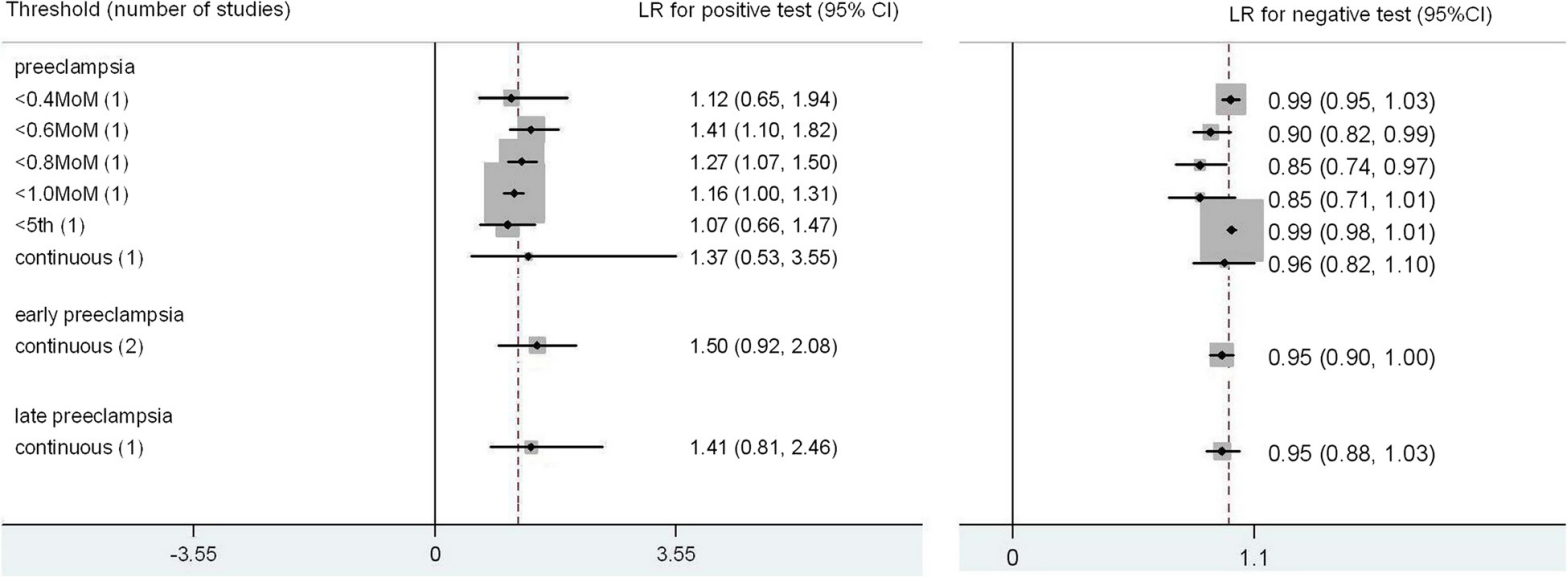
Forest plot for hCG to predict preeclampsia: forest Plot showing likelihood ratio of a positive and negative test result with 95 % confidence intervals (95 % CI) for studies of Human chorionic gonadotrophin (hCG) to predict preeclampsia. * continuous: Likelihood ratio calculated from receiver operating curve analysis

The results for hCG to predict preeclampsia are summarized in Fig. 8. The total number of women in these studies is 112,400. There were 4 included studies in the meta-analysis [7, 10, 11, 18], and there was single study for every threshold. The most accurate predictor was hCG < 0.6MoM; LR+ 1.41 (1.10, 1.82), LR- 0.90 (0.82, 0.99), sensitivity 28 % (2 %, 71 %), specificity 80 % (36 %, 99 %). There were 2 studies and 1 study looking at early preeclampsia [18, 19] and late preeclampsia [19] respectively as the outcome, results showed no improvement in prediction.

**Fig. 9:**
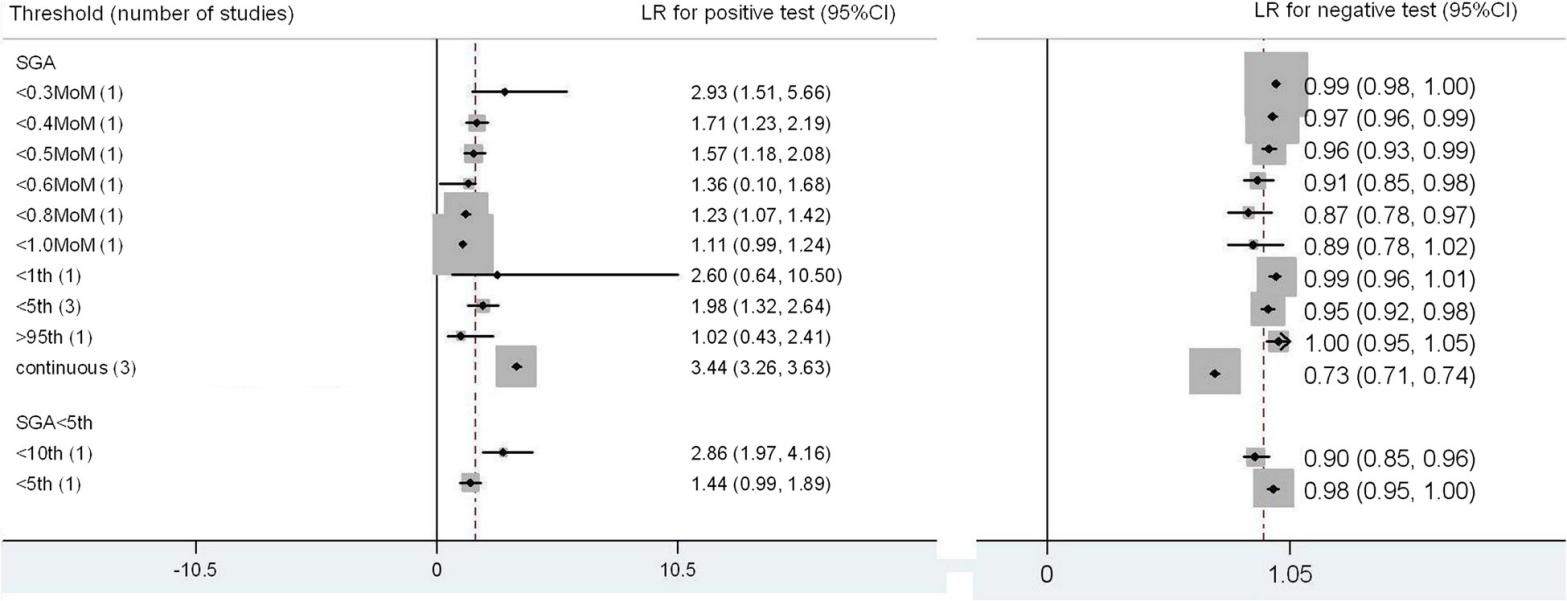
Forest plot for hCG to predict SGA: forest Plot showing likelihood ratio of a positive and negative test result with 95 % confidence intervals (95 % CI) for studies of Human chorionic gonadotrophin (hCG) to predict small for gestational age (SGA). * continuous: Likelihood ratio calculated from receiver operating curve analysis

For SGA < 10^th^ centile there were 9 included studies in the meta-analysis [7, 11, 23–25, 29, 30, 35, 36] (Fig. 9). The commonest thresholds used were hCG < 5^th^ (3 studies) and calculated from receiver operating curve analysis. The most accurate predictor for SGA < 10^th^ centile was calculated from receiver operating curve analysis; LR+ 3.44 (3.26, 3.63), LR-0.73 (0.71, 0.74), sensitivity 34 % (32 %, 37 %), specificity 90 % (89 %, 91 %). For SGA < 5^th^ centile there were only 2 thresholds studied with single study for each[7, 10], hCG < 10^th^ centile was more accurate; LR+ 2.86 (1.97, 4.16), LR-0.90 (0.85, 0.96), sensitivity 15 % (5 %, 26 %), specificity 95 % (87 %, 100 %).

**Fig. 10:**
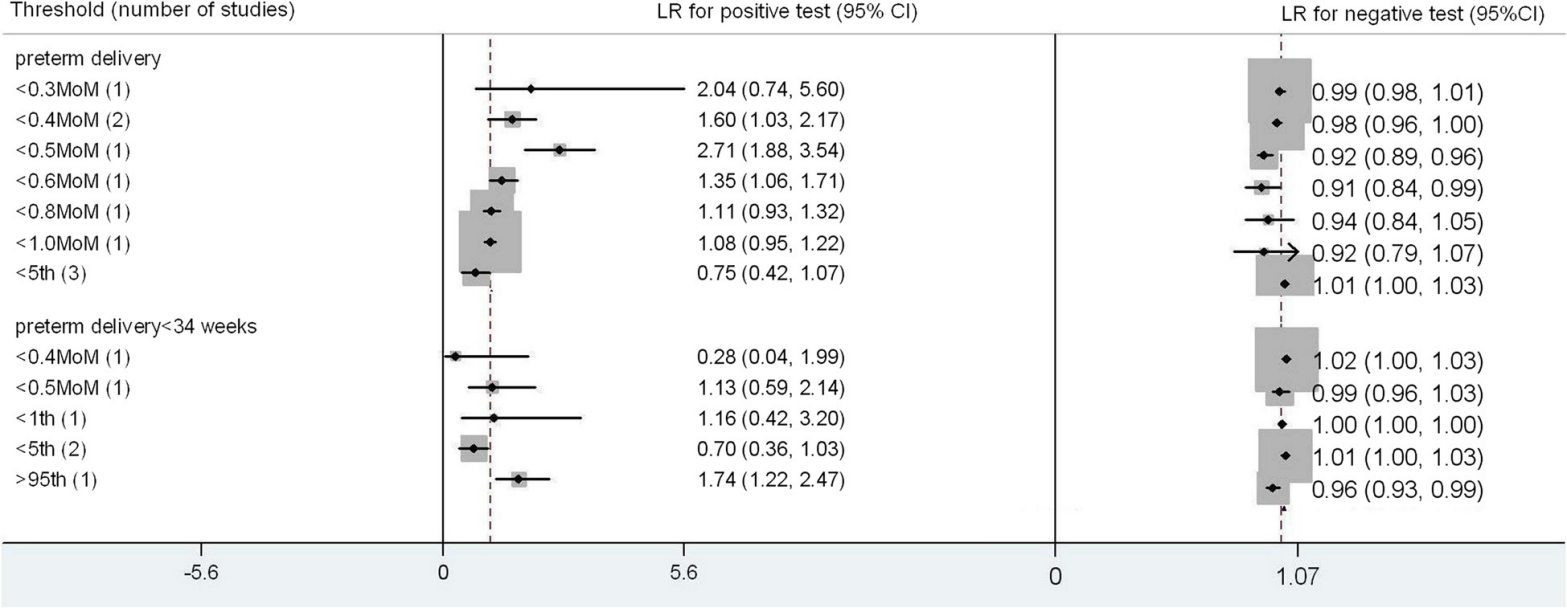
Forest plot for hCG to predict prelivery: forest Plot showing likelihood ratio of a positive and negative test result with 95 % confidence intervals (95 % CI) for studies of Human chorionic gonadotrophin (hCG) to predict preterm delivery

There were 6 studies for preterm delivery [7, 10, 11, 23, 35, 40] and 4 studies for preterm delivery < 34 weeks [7, 23, 25, 40] included in the meta-analysis (Fig. 10). The commonest threshold used to predict preterm delivery were hCG < 5^th^ centile (6 studies). The best predictor for preterm delivery was hCG < 0.5 MoM; LR+ 2.71 (1.88, 3.54), LR- 0.92 (0.89, 0.96), sensitivity 12 % (5 %, 21 %), specificity 96 % (89 %, 98 %). For preterm delivery < 34 weeks, hCG > 95^th^ was the most accurate predictor, LR+ 1.74 (1.22, 2.47), LR-0.96 (0.93, 0.99), sensitivity 9 % (2 %, 29 %), specificity 95 % (76 %, 99 %).

## Discussion

We evaluated the accuracy of five serum screening markers commonly used in first trimester screening for preeclampsia, SGA and preterm delivery. The results showed low predictive accuracy overall. For preeclampsia, the best predictor was PlGF. However, it is important to point out that this threshold was determined from a receiver operating characteristic curve and based only 2 studies. For early and late preeclampsia, the best predictor was also PlGF. Generally, the predictive value of serum markers for early preeclampsia is better than that of late preeclampsia. For SGA the best predictor overall was PP13 while PAPPA < 1^st^ centile was the best predictor of SGA < 5^th^ centile. These results were both based on single studies. For preterm delivery, the best predictor was PP13 while PAPPA < 0.3 MoM was the best predictor of preterm delivery < 34 weeks.

The predict value of first trimester analytes is not worse compare to that of the second trimester markers. Previous studies show in the second trimester, the most accurate predictor of hCG for preeclampsia was hCG > 2.0 MoM, with LR+ 2.45 (1.57, 3.84), LR- 0.89 (0.83, 0.96); for SGA was hCG > 2.0 MoM, with LR+ 1.74 (1.48, 2.04), LR-0.95 (0.93, 0.96). The most accurate predictor of PAPPA for preeclampsia was PAPP-A < 5^th^ centile, with LR + 2.10 (1.57, 2.81), LR- 0.95 (0.93, 0.98); for SGA was PAPP-A < 1^st^ centile; LR+ 3.50 (2.53, 4.82), LR- 0.98 (0.97, 0.99). On the other hand, our meta-analysis shows the most accurate predictor of hCG for preeclampsia was hCG < 0.6 MoM; LR+ 1.41 (1.10, 1.82), LR- 0.90 (0.82, 0.99), for SGA was calculated from receiver operating curve analysis; LR+ 3.44 (3.26, 3.63), LR-0.73 (0.71, 0.74). The most accurate predictor of PAPPA for preeclampsia was PAPPA < 0.4 MoM; LR+ 2.17 (1.48, 3.17), LR- 0.91 (0.85, 0.97), for SGA was PAPPA < 1^st^ centile; LR+ 3.59 (2.77, 4.40), LR- 0.98 (0.97, 0.98). A possible explanation for the apparent difference of hCG change between first trimester and second trimester is that the low levels at first trimester are the consequence of impaired placentation and smaller placental mass, whereas the high levels in the second trimester may be the result of ‘leakage’ or hypoperfusion-related stimulation of production of this hormone [47]. Although the symptoms of preeclampsia and FGR generally manifest in the second to third trimester of pregnancy, their underlying pathology takes place in the first trimester. One possible reason why preventive strategies have proven very disappointing at present is that the proposed interventions have commenced in the mid to late second trimester, when the underlying placental dysfunction may already be established. Earlier assessment before the establishment of placental dysfunction may have the potential to improve predictive value for clinical practice. With the increased use of first-trimester screening for Down syndrome, there is the opportunity to ‘piggy back’ screening tests for preeclampsia, FGR and preterm delivery onto existing tests.

As preeclampsia and SGA are diseases with relatively low prevalence, a clinically useful test would need to have a high positive LR (> 10) and low negative LR (< 0.10) [48]. From the results of this review it is unlikely that any first trimester serum screening marker in isolation will provide this. Future research should thus concentrate in two areas. The first is to improve the knowledge of the biological mechanisms for the abnormal clinical tests by focusing on the exact placental pathology resulting in the changes seen in preeclampsia, FGR and preterm delivery. Preliminary findings suggest that genomic studies can improve our understanding of the early pathophysiology of preeclampsia/FGR/preterm delivery at the molecular level. It is hoped that proteomics, metabolomics, and other techniques will allow us to provide potential targets for the development of biomarkers with high enough predictive and prognostic information to be translated into clinical practice. Secondly, future research should attempt to improve the predictive value by combining Doppler sonography, different maternal serum analytes and clinical characteristics. The use of multiple parameters increases the specificity and sensitivity of the screening possibly because they reflect different pathways to the disease process, with abnormal Doppler reflecting the inadequate trophoblastic invasion of the maternal spiral arteries and abnormal biomarkers demonstrating the dysregulated secretory activity by the trophoblasts. However, some studies showed no additive effect of combining different markers, likely secondary to correlation between the markers (such as ADAM12 and PAPP-A, sFlt-1 and sEng) [47]. Sequential measurements of markers might also improve the risk assessment as individual changes from the first to second trimesters have been shown to occur in preeclampsia and FGR.

Our result also showed the detection rate of first trimester serum markers for early preeclampsia is better than that for late preeclampsia. This disparity may result from different etiologies between early and late preeclampsia. Early preeclampsia is said to be associated with inadequate and incomplete trophoblast invasion of maternal spiral arteries, and is often complicated with a fetal growth restriction. In contrast, the late onset type of preeclampsia is often related to enlarged placental mass or surface (diabetes, multiple pregnancies, anemia, high altitude). It often shows normal or only slightly altered behavior of the uterine spiral arteries and thus no changes in the blood flow of the umbilical arteries. Fetus with late onset preeclampsia often shows no signs of any growth restriction [49]. Since abnormal concentration of serum markers in the first trimester is caused by intrinsic alteration of the villous trophoblast, it is reasonable that predictive value would be poorer for late onset preeclampsia with normal or only slightly altered trophoblast invasion in the first trimester.

The strength of the study includes generally sufficient quality and a quality assessment of studies based on recognized criteria. However, there are still some limitations. First, there is large discordance in reports of cutoff points, thus, a formal meta-analysis with estimated overall relative risks was not feasible. Secondly, the number of studies for some cutoffpoints is so small that they lead to some contradictive results. For example, our analysis shows the best predictor for preterm delivery was hCG < 0.5 MoM while the best predictor for preterm delvery < 34 weeks is a hCG > 95^th^ centile. This odd result is probably due to the small numbers of studies since there is only one study for each threshold. Clearly large scale studies are needed for more reliable evaluation. Thirdly, all of the studies we selected are population of low risk so we are unable to perform a sub analysis. We didn’t choose the population of high risk since there are few studies on it. More studies are needed to analyse predictive accuracy by the type of population.

## Conclusion

First trimester screening analytes have low predictive accuracy for pre-eclampsia, SGA and preterm delivery. However, the predict value of first trimester analytes is not worse compare to that of the second trimester markers. They may be useful in prediction when combined with other tests. Early pathophysiology of preeclampsia/FGR/preterm delivery should be studied to develop biomarkers with high enough predictive and prognostic information to be translated into clinical practice.

## Abbreviations

PAPP-A: Pregnancy- associated plasma protein-A
hCG: Human chorionic gonadotropin
PlGF: Placental growth factor
PP13: Placental protein 13
SGA: Small for gestational age
LR: Likelihood ratio
